# Smooth muscle cell specific deletion of the PKGIα target RGS2 induces vascular dysfunction and hypertension

**DOI:** 10.1186/1471-2210-11-S1-P72

**Published:** 2011-08-01

**Authors:** Robrecht Thoonen, Haihua Zhang, Yuichi Abe, Heather Nickerson, Mark Aronovitz, Pierre Chambon, Richard H Karas, Michael E Mendelsohn

**Affiliations:** 1 Molecular Cardiology Research Institute, Tufts Medical Center, Boston, Massachusetts, USA; 2 IGBMC, CNRS/INSERM/ULP, Collège de France, Illkirch Cedex, France; 3 Present address, Merck Research Laboratories, Rahway, NJ, USA

## Introduction

Current dogma regarding the etiology of hypertension states that renal abnormalities of sodium handling cause an increase in blood volume that overwhelms vascular counter-regulatory responses, resulting in hypertension. Although it is known that acute changes in vascular morphology and tone can cause an increase in vascular resistance and blood pressure, we have hypothesized that primary abnormalities of the regulation of vascular smooth muscle tone can also be an etiology for hypertension. We have previously demonstrated that disruption of the leucine zipper targeting domain in PKGIα results in dysregulation of vasomotor tone and hypertension in the setting of normal renal function. To directly test the hypothesis that abnormalities in vasomotor regulation can be the primary etiology of hypertension, we have developed and now characterize a novel mouse model harboring a smooth muscle cell (SMC) specific deletion of the PKGIα target we identified previously, RGS2, a GTPase activating protein that inhibits Gq-coupled G-protein coupled receptor (GPCR) signaling, whose total body deletion in mice causes hypertension (Tang et al, Nature Medicine, 2003).

## Results

Smooth muscle specific RGS2 knockout mice (RGS2-SMC-KO) were generated by crossing mice carrying an RGS2 gene with exons 2 to 5 flanked by LoxP sites with inducible SMA-CRE-ER^T2^ mice. SMC-specific deletion of RGS2 was induced by tamoxifen administration at the age of 7 weeks. Cre negative mice treated with tamoxifen were used as controls. Telemetric monitoring (DSI) of blood pressure (BP) and heart rate (HR) in male RGS2-SMC-KO mice showed that these mice have a significant increase in systolic BP vs. control mice (135 ± 5 mmHg vs. 122 ± 7 mmHg, respectively. *P*<0.01) without a concomitant change in HR. The change in BP after challenge with a bolus injection of the NOS inhibitor L-NAME (50 mg/kg i.v.) was significantly less in the RGS2-SMC-KO mice compared to WT mice. (ΔSBP: 38 ± 7 mmHg vs. 47 ± 3 mmHg, respectively. *P*<0.001), consistent with decreased basal endogenous NO signaling. BP responses to changes in dietary sodium intake in the RGS2-SMC-KO mice were identical to control mice.

## Conclusion

Though further characterization of renal function and vasomotor regulation in RGS2-SMC-KO mice is ongoing, the current data demonstrate the presence of hypertension in these mice, together with normal sodium handling, supporting the hypothesis that high blood pressure can arise from a primary abnormality of vascular smooth muscle cell contractile regulation. These findings, taken together with the diminished SBP response to L-NAME, support that endogenous NO signaling, and the activation of the NO-sGC-PKGI pathway in smooth muscle cells, are necessary to maintain normal vascular tone and blood pressure by attenuating Gq mediated GPCR constrictive signaling.

